# Supplementary material for: Chromothripsis during telomere crisis is independent of NHEJ, and consistent with a replicative origin
Source: Genome Res. 2019 May;29(5):737–49. doi: 10.1101/gr.240705.118 (PMC6499312; doi:10.1101/gr.240705.118)
Supplement: Supplemental Material [file supp_gr.240705.118_Supplemental_file_1.zip › contigs/annotated_contigs/DB98/contig.4.DB98_length_624_mean_cov_12.3701923077.docx]

**DB98_length_624_mean_cov_12.3701923077**

```
TATTCTTGAAATTGGTTGTCTTTAATTTCTTTAAGTTAATTATATTTATTTAATGTGGCTCATCCAAATTTAAGCTTCTTGAATGTAAG
 >chr3:104261131-104261424 - E=1e-158
AAGGATGCTTTATTGTTAAAAATTTTTGGAACTGCATTTTGCCAAAGTTCCAAATGCAGTGCTGGAGATACAGGTATTGCCAATAATAG

CTTCTTATTTGACTAAATCTATTTCTAAATTACAATTTGTTTAGTGTATTCTAAAATGTGATTTTTTTCATCATAAGAGTAGCTGGTTC

TGCACCTTATTTTAAGGAAATTATAT|AAATATATAAATAAATAAATAAATAAATAAATAATATATAAATAAATAAATAA|TATATAAA
                            >chr16:85747988-85748041 - E=4e-14 p=0e+00           >chr5:
TAATATATAAATAATATATAAATAATATTTTATATAT|TTTAAGATAAATATATAAA|TAATATTTATCTATAACATGCAATGCTGTTT
105410984-105411029 + E=3e-03 p=0e+00                    >chr3:105485077-105485291 - E=
GATAATATTTTACTCACAATAGAGTTTCATTCAAAATTGGAGTCAGTCCTCTCAAACCCTGCCACTGCTTTATCAACCAAGTGTCTGCA
5e-117
ATATTCTAAATCCTTTATTGTAATATCAGCAGTGTTCACAGCATCTTCACCTGGAGTAAATTACATTTAAGAAAGCACTTTTTTGCTTT

TCTAT
```
